# Text mining resources for the life sciences

**DOI:** 10.1093/database/baw145

**Published:** 2016-10-25

**Authors:** Piotr Przybyła, Matthew Shardlow, Sophie Aubin, Robert Bossy, Richard Eckart de Castilho, Stelios Piperidis, John McNaught, Sophia Ananiadou

**Affiliations:** 1 National Centre for Text Mining, School of Computer Science, University of Manchester, Manchester, UK; 2 Institut National de la Recherche Agronomique, Jouy-en-Josas, France; 3 Ubiquitous Knowledge Processing Lab, Technische Universität Darmstadt, Darmstadt, Germany and; 4 Institute for Language and Speech Processing, Athena Research Center, Athens, Greece

## Abstract

Text mining is a powerful technology for quickly distilling key information from vast quantities of biomedical literature. However, to harness this power the researcher must be well versed in the availability, suitability, adaptability, interoperability and comparative accuracy of current text mining resources. In this survey, we give an overview of the text mining resources that exist in the life sciences to help researchers, especially those employed in biocuration, to engage with text mining in their own work. We categorize the various resources under three sections: *Content Discovery* looks at where and how to find biomedical publications for text mining; *Knowledge Encoding* describes the formats used to represent the different levels of information associated with content that enable text mining, including those formats used to carry such information between processes; *Tools and Services* gives an overview of workflow management systems that can be used to rapidly configure and compare domain- and task-specific processes, via access to a wide range of pre-built tools. We also provide links to relevant repositories in each section to enable the reader to find resources relevant to their own area of interest. Throughout this work we give a special focus to resources that are interoperable—those that have the crucial ability to share information, enabling smooth integration and reusability.

## Introduction

Text mining empowers the researcher to rapidly extract relevant information from vast quantities of literature. Despite the power of this technology, the novice user may find text mining unapproachable, with an overload of resources, jargon, services, tools and frameworks. The focus of this article, and one of the obstacles that have limited the widespread uptake of text mining, is a lack of specialist knowledge about text mining among those researchers who could most benefit from its results. Our contributions in this article are intended to inform and equip these researchers such that they will be better placed to take full advantage of the panoply of resources available for advanced text mining in the life sciences. In this context, *resources* refers to anything that could help in such a process, such as annotation formats, content sharing mechanisms, tools and services for text processing, knowledge bases and the accepted standards associated with these.

As of 2016, PubMed, the most widely used database of biomedical literature, contains over 26 million citations^1^. It is growing constantly: over 800 000 articles are added yearly^2^ and this number has substantially increased over the previous few years (1). This constant increase is recognized as a major challenge for evidence-based medicine (2), as well as other fields (3). One of the tools for tackling this problem is text mining (TM). However, as many surveys have shown (4–6) the TM landscape is fragmented and, at its worst, it can be hostile to the uninitiated, giving rise to such questions as: Where should I look for resources? How should I assemble and manage my TM workflows? How should I encode and store their output? How can I ensure that others will be able to use the TM outputs I wish to share from my research? Moreover, one may be drawn to popular standards—while lesser known standards go unnoticed, yet may be more suitable. When taking stock of the current literature, we identified three key areas which we address through this article. First, there is a lack of aggregated knowledge sources, entailing search through dozens of separate resources to find those that are appropriate to one’s work. We have addressed this need by providing numerous tables that give an overview of key resources throughout this work. At the end of each section we also provide a table of repositories that can be browsed to find further resources of interest to the reader. Second, we found that there was no clear outline of the text mining process in the literature. The structure of this survey follows the text mining process, beginning with content discovery, moving to annotation formats and ending with workflow management systems that enable text mining in the life sciences. Third, we found a lack of focus on interoperability, i.e. ability of resources to share information and cooperate, which is achieved by using widely accepted standards. Although the interoperability issue is known to most researchers, not enough is done in the literature to promote interoperable resources to the communities who may benefit from them. Interoperability was named as one of the major obstacles when implementing text mining in biocuration workflows (7). We have placed a high focus on interoperability throughout this report, suggesting where and when interoperable resources can be used. Interoperability is not appropriate for every task, but we take the view that in these cases the user should know about the interoperable options and make a conscious choice.

Interoperability is vital at multiple levels of granularity, such as the way that a TM tool encodes input and output (8–10); the format of metadata that is associated with a resource in a repository (11); or the licences associated with software and content. Each of these levels must be addressed if we wish to promote a culture of true interoperability within TM. We have addressed several levels that are relevant to the text-mining life scientist as seen through the sections of this report.

This paper has been split into the following sections to categorize and help understand the existing technologies:

- In ‘Content discovery’ section, we start by explaining how and where to get the input text data, i.e. corpora of publications. We describe metadata schemata and vocabularies and discuss the most popular publication repositories.
- In ‘Knowledge encoding’ section, we show how the original document is encoded with additional information, describing annotation formats. We also outline formats for knowledge resources that are frequently employed in the TM process and describe a few examples of such databases, especially from the area of life sciences. We end by discussing content repositories that may be of use to the reader.
- In ‘Tools and services’ section, we look at methods of annotating and transforming the acquired data, including software tools, workflow engines and web services. We also describe repositories that let users easily discover such resources.
- Finally, in ‘Discussion’ section, we discuss the landscape described in previous sections, focusing on interoperability and its importance for TM in the life sciences.

Since the subject matter described in this survey is vast, the reader may wish to initially explore those parts that are pertinent to their own research. Biocurators with no background in text mining may wish to begin with points 2.4, describing repositories of publications; ‘Annotation models’ section explaining annotation formats; the ‘Useful knowledge resources’ section including most popular knowledge resources and ‘Text mining workflow management systems’ section introducing text mining workflow systems. Nevertheless, both biocurators and text mining experts should be able to take value from reading the survey as a whole. We hope that as the novice biocurator grows in their knowledge of the field, both through reading this report and other materials that they will come to treat the information herein as a useful point of reference. We have categorized the information into structured tables throughout to help the reader quickly find and compare the information that they seek.

## Content discovery

In this section, we will explore how users can access and retrieve publications that are relevant to their research. Although many other document types can be mined (e.g. Electronic Health Records, Patent Applications or Tweets), in this work we have focused on scholarly publications. This is because there is a large amount of information to be mined from such literature, making it a very good starting point in most fields. Many of the resources and services described in this article can be easily transferred to other types of content. We explain and discuss *repositories*, and *aggregators*, *metadata*, *application profiles* and *vocabularies.* We mention several web-accessible repositories, aggregators and their features, which are considered interesting and useful for the life sciences researcher.

Publications are usually stored in searchable structured databases typically called *repositories*. Although many repositories stand alone, an aggregator may connect several repositories in looser or tighter networks by aggregating publications, or information about them, from other repositories in the network. The internal mechanism of a repository relies on a set of structured labels, known as metadata. Metadata can generally be defined as data used to describe data, and as such metadata may themselves be stored and managed in repositories usually called metadata repositories (or registries, or simply catalogues). Usually, aggregators act as metadata repositories in that they harvest metadata from repositories and make them available to facilitate the search and discovery of publications. Metadata for scientific articles, e.g. should include authors' names and affiliations, date of publication, journal or conference name, publisher, sometimes scientific domain or subdomain, etc., in addition to article title and an appropriate identifier. As the metadata needs of particular applications or scientific communities may vary, metadata can be combined into application profiles. Application profiles specify and describe the metadata used for particular applications, including, e.g. refinements in the definitions as well as the format and range of values permitted for specific elements. Usually, aggregators design and make use of application profiles. We particularly focus on the format and vocabulary of the metadata used in repositories. Without a proper understanding of the operational principles of the repositories and/or aggregators, and the metadata they use to document their content, users may struggle to retrieve publications.

There is a wide range of metadata schemata and application profiles used for the description of content and resources. This variety is, to a great extent, due to the diverse needs and requirements of the communities for which they are developed. Thus, schemata for publications originally came from publishers, librarians and archivists. Currently, we also witness the cross-domain activities of the various scientific communities, as the objects of their interest expand to those of the other communities, e.g. in order to link publications of different domains, publications and the supplementary material described in them, or services which can be used for processing publications and/or other datasets.

Differences between the schemata are attested at various levels such as:

- types of information (e.g. identification, resource typing, provenance, classification, licensing, etc.) covered by the schema;
- the granularity of the schema, ranging from detailed schemata to very general descriptions, including mandatory, recommended and optional description elements;
- degree of freedom allowed for particular elements (e.g. use of free text statements vs. recommended values vs. entirely controlled vocabularies)
- use of alternative names for the same element(s) or use of the same name with different semantics.

All of the above features, especially the degree of granularity and control of the element values, influence the discoverability of publications via their metadata and, in consequence, the applicability and performance of the TM process.

### Metadata schemata and profiles

To make publications and other types of content, data and services discoverable we use metadata. Metadata will enable the biocurator to search repositories and aggregators for content that is appropriate for his/her purposes using specific metadata elements or filtering the retrieved results (i.e. publications, language and knowledge resources or TM tools and services) using specific values of each metadata element. So, e.g. a biocurator wishing to compile a collection of publications can search a repository or aggregator for publications from a certain publishing body, in a particular language and topic, while he can further filter the retrieved results for those that are available under open access rights. This section presents the most common metadata schemata and application profiles used for the description of publications in the life sciences domain. In order to make metadata information interoperable, we use schemata that define common encoding formats and vocabularies (Table 1).

**Table 1.** A comparison of popular metadata schemata, used to encode information about publications

Name	Last updated	Domain	Main use
Dublin Core (DC)/DC Metadata Initiative (DCMI)^a^	June 2012	Generic	Widely accepted standard
Journal Article Tag Suite (JATS)^b^	Actively Maintained	Journal Articles	Open access journals
DataCite^c^	Actively Maintained	Research Data and Publications	Citations
CrossRef^d^	Actively Maintained	Research Data and Publications	Citations
BibJSON^e^	Actively Maintained	Bibliographic information	Bibliographic metadata
CERIF^f^	Actively Maintained	Research Information	European research
CKAN^g^	Actively Maintained	Generic	Data management portals

Different formats describe different types of items as shown in the ‘Domain’ and ‘Main Use’ columns.

a http://dublincore.org/

b https://jats.nlm.nih.gov/

c https://www.datacite.org/

d http://www.crossref.org/

e http://okfnlabs.org/bibjson/

f http://www.eurocris.org/cerif/main-features-cerif

g http://ckan.org/

Dublin Core (12) is a widely used metadata schema, best suited to resource description in the general domain. It consists of 15 generic basic elements used for describing any kind of digital resource. DCMI Metadata Terms consist of the full set of metadata vocabularies used in combination with terms from other compatible vocabularies in the context of application profiles. For many metadata schemata, there are mappings to DC elements for metadata exchange. DC is often criticized as being too minimal for expressing more elaborate descriptions required by specific communities. To remedy this defect, DC is usually extended according to DCMI specifications.

JATS (13) is a suite of metadata schemata for publications, originally based on the National Library of Medicine (NLM) Journal Archiving and Interchange Tag Suite. The most recent^3^, defines a set of XML elements and attributes for tagging journal articles both with external (bibliographic) and internal (tagging the actual textual content of an article) metadata. In the life sciences area, DC and JATS are supported by PubMed Central^4^ (PMC) as formats for metadata retrieval and harvesting, as well as for authoring, publishing and archiving.

DataCite (14) represents an initiative for a metadata schema, along the same lines as JATS, aspiring to cover all types of research data, while it is more tuned to metadata-based description of publications. It places a strong emphasis on citation of research data in general, not only including publications, and for this reason it strongly supports the use of persistent identifiers in the form of digital object identifiers (DOIs, see ‘Mechanisms used for the identification of resources’ section). Similarly, CrossRef (15) is a registry for scholarly publications, stretching out to research datasets, documented with basic bibliographic information and heavily also relying on DOIs for citation and attribution purposes. BibJSON is a convention for representing bibliographic metadata in JSON facilitating the sharing and use of such metadata.

The Comprehensive Knowledge Archive Network (CKAN) (16) is essentially an open data management software solution, very popular among the public sector open data communities. Intending to be an inclusive solution, CKAN features a generic, albeit limited, set of metadata elements covering many types of datasets. Similarly, Common European Research Information Format (CERIF) (17) proposes a data model catering for the description of research information and all entities and relationships among them (researchers, publications, datasets, etc.)

Building on metadata schemata, many initiatives have defined their own application profiles. As indicated in the previous section, application profiles specify the metadata terms that an information provider uses in its metadata, identify the terms used to describe a resource and may also provide information about term usage by specifying vocabularies or other restrictions on potential values for metadata elements; they may go further to describe policies, as well as operational and legal frameworks. OpenAIRE^5^, for example, has proposed and used an application profile and harvests metadata from various sources, notably repositories of scholarly publications in OAI_DC format, data archives in DataCite format, etc., while they are currently considering publishing homogenized OpenAIRE metadata as Linked Open Data (LOD). RIOXX^6^ is a similar application profile targeting mainly open access repositories in the UK. It is also based on DC with references to other vocabularies, like JAV^7^, while adhering to many of the OpenAIRE guidelines.

#### Metadata schemata and profiles for language and knowledge resources

Text mining processes are closely related to language and knowledge resources that are either used as conceptual reference material for annotating text (e.g. scientific publications) or as resources for the creation and operation of text mining tools and services. Language/knowledge resources have been, in the past three decades, recognized as the raw materials for language processing technologies and as one of the key strands of text mining research and development. In order to cover both the varieties of language use and the requirements of linguistic research, several initiatives have proposed metadata schemata for documenting language resources. Using such metadata, biocurators can search repositories and aggregators for vocabularies, terminologies, thesauri, corpora made up of (annotated) scientific publications or other types of content as well as text mining tools and services pertinent to the life sciences domain. Table 2 lists some of the most widespread of these schemata.

**Table 2.** A comparison of metadata schemata used for documenting language resources

Name	Last Updated	Domain	Main use
TEI^a^	Actively Maintained	Documents	Encoding text corpora
CMDI^b^	Actively Maintained	Generic	Infrastructure for metadata profiles
META-SHARE^c^	Actively Maintained	Language Resources	Metadata schema for language resources and services documentation
LRE Map^d^	Updated at each LREC conference (biennial)	Language Resources	Metadata schema for language resources

a http://www.tei-c.org/

b http://www.clarin.eu/content/component-metadata, http://www.clarin.eu/ccr/

c http://www.meta-net.eu/meta-share/metadata-schema, http://www.meta-share.org/portal/knowledgebase/home

d http://www.resourcebook.eu/searchll.php

The Text Encoding Initiative (TEI) (18) represents a ‘standard for the representation of texts in digital form’, currently the most widely used format in the area of the humanities. To some extent similarly to JATS, the TEI P5 guidelines^8^ include recommendations both for the bibliographic-style description of texts as well as for the representation of the internal structure of the texts themselves (form and content) and their annotations.

The Common Language Resources and Technology Infrastructure (CLARIN) Research Infrastructure (19) has proposed CMDI, a flexible mechanism for creating, storing and using various metadata schemata, in an attempt to accommodate the diverse needs of language technology and text mining research and to promote interoperability. Along the same lines, the META-SHARE metadata schema (11) is used in the META-SHARE infrastructure (20) to describe all kinds of language resources including datasets (e.g. corpora, ontologies, computational lexica, grammars, language models, etc.) and language processing tools/services (e.g. parsers, annotators, term extractors, etc.). A subset of these metadata components is common to all resource types (containing administrative information, e.g. contact points, identification details, versioning, etc.), while metadata referring to technical information (e.g. text format, size and language(s) for corpora, requirements for the input and output of tools/services, etc.) are specific to each resource type.

Finally, the LRE Map (21) features a minimal, yet practical for its purposes, metadata schema that is used for crowdsourcing metadata information for language resources, including datasets and software tools and services, directly by authors who submit their publications to the LREC Conferences^9^.

To facilitate interoperability between metadata schemata and the repositories that use them, including those described above, the World Wide Web Consortium (W3C) has published the Data Catalog (DCAT) Vocabulary^10^. DCAT is an RDF vocabulary catering for the description of *catalogues*, *catalogue records*, their *datasets* as well as their forms of *distribution*, e.g. as downloadable file, as web service that provides the data, etc. DCAT is now extensively used for government data catalogues and is also growing in popularity in the wider Linked Data community.

### Vocabularies and ontologies for describing specific information types

Metadata schemata are not enough for comprehensive description of resources, as we also need to know what particular fields mean. For example, different metadata schemata for scientific articles may include a field called ‘subject’, or ‘domain’ but this raises questions: Are ‘subject’ and ‘domain’ intended to codify the same information? Are the values for these fields provided freely by the authors, or do they have to be selected from a controlled vocabulary or an ontology? Such questions are usually addressed when designing application profiles where *inter alia* vocabularies and/or ontologies associated with particular fields are specified. The resources in Table 3, mainly controlled vocabularies, authority lists and ontologies, are presented because they are used widely and can be useful for improving existing schemata in recording information.

**Table 3.** A comparison of vocabularies and ontologies for metadata description, used in conjunction with metadata schemata to give meaningful descriptions of resources

Title	Domain	Format
Medical Subject Headings (MESH)^a^	Medicine	XML
EDAM (EMBRACE Data and Methods) ontology^b^	Bioinformatics	OWL, OBO
Dewey Decimal Classification (DDC)^c^	Library classification	–
Universal Decimal Classification (UDC)^d^	Library classification	–
Library of Congress Subject Headings (LCSH)^e^	Library classification	–
EuroVoc^f^	Document classification	XML, SKOS/RDF
Semantic Web for Research Communities (SWRC)^g^	Research communities	OWL
CASRAI dictionary^h^	Research administration information	HTML
Bibliographic Ontology (BIBO)^i^	Bibliographic information (citations and bibliographic references)	RDF/RDFS
COAR Resource Type Vocabulary^j^	Open access repositories of research outputs	SKOS
PROV Ontology (PROV-O)^k^	Provenance information	OWL2
Open Digital Rights Language (ODRL)^l^	Digital Rights Management, Licensing	RDF/XML
Creative Commons Rights Expression Language (ccREL)^m^	Intellectual Property Rights, Digital Rights Management, Licensing	RDF

A wide variety of formats and sizes, suitable for different domains, is reported above. Although it is difficult to compare size due to different formats, we have presented the resources in approximate order of the number of items held in each at the time of writing from most to least.

a https://www.nlm.nih.gov/mesh/

b http://edamontology.org/page

c https://www.oclc.org/dewey.en.html

d http://www.udcc.org/index.php/site/page?view=about

e http://id.loc.gov/authorities/subjects.html

f http://eurovoc.europa.eu/

g http://ontoware.org/swrc/

h http://dictionary.casrai.org/Main_Page

i http://bibliontology.com/

j https://www.coar-repositories.org/

k https://www.w3.org/TR/prov-o/

l https://www.w3.org/ns/odrl/2/ODRL21

m https://wiki.creativecommons.org/wiki/CC_REL

The vocabularies of Table 3 represent variably structured conceptualizations of different aspects in the lifecycle of a resource (or in general of a content item) from basic bibliographic description to its reuse and associated intellectual property and distribution rights.

Focusing on the medical domain, Medical Subject Headings (MeSH) (22) is one of the most widely used controlled vocabularies for classification, and EDAM (EMBRACE Data and Methods) (23) is an ontology of well established, familiar concepts that are prevalent within bioinformatics, including types of data and data identifiers, data formats, operations and topics.

A range of controlled vocabularies that have evolved from flat lists of concepts into hierarchical classification systems or even full-fledged ontologies are employed for standardizing, to the extent possible, subject domain classes. Springing from library sciences as well as documentation and information services, the Dewey Decimal Classification (DDC) (24), the Universal Decimal Classification (UDC) (25) and the Library of Congress Subject Headings are among the most widely used systems for the classification of documents and collections. EuroVoc is a similar system, represented as a thesaurus covering the activities of the European Parliament, and gradually expanding to public sector administrative documents in general. EuroVoc is multidisciplinary, as is the case for the previously mentioned classification systems, enriched however with a strong multilingual dimension in that its concepts and terms are rendered in all official languages of the EU (and those of 3 EU accession countries), thus paving the way for cross-lingual interoperability.

The Semantic Web for Research Communities (SWRC) is a generic ontology for modelling entities of research communities such as persons, organizations, publications and their relationships (26), while the Bibliographic Ontology (BIBO) caters mostly for bibliographic information providing classes and properties to represent citations and bibliographic references. COAR (27) is a controlled vocabulary, described in SKOS (a popular format for encoding thesauri, see ‘Formats for knowledge resources’ section), for types of digital resources, such as publications, research data, audio and video objects, etc. The PROV Ontology (PROV-O), a W3C recommendation, provides a model that can be used to represent and interchange provenance information generated in different systems and under different contexts.

Finally, catering for expressing information about rights of use, reuse and distribution, the Creative Commons Rights Expression Language (ccREL) (28) formalizes the vocabulary for expressing licensing information in RDF and the ways licensing may be attached to resources, while Open Digital Rights Language (ODRL) (29) provides mechanisms to describe distribution and licensing information of digital content.

Using these vocabularies, biocurators and text mining researchers can effectively search and retrieve content from digital repositories and also use them to annotate content and data both externally (e.g. tag a document or collection) and internally (e.g. annotate text spans as referring to a certain concept in an ontology or term in a vocabulary).

### Mechanisms used for the identification of resources

Identification systems present the researcher with a means of assigning a persistent identifier to a resource (usually under their ownership). In contrast to simple identifiers, a persistent identifier is actionable on the Web and can be distributed to other researchers who can also use the same identifier to refer to the original resource. This facilitates deduplication, versioning and helps to indicate the relation between resources (e.g. raw and annotated text, scholarly articles before and after review, corrected or enriched versions of a lexicon, etc.). Although persistent identifiers have so far been assigned primarily to publications, they are recently also applied elsewhere: e.g. datasets, software libraries or even the individual researcher. Below, we present the main mechanisms used for assigning Persistent Identifiers (PIDs). Similarly to persistent URL solutions (permalink and PURL^11^), the assignment of unique PIDs allows one to refer to a resource throughout time, even when it is moved between different locations. Some of the most popular PID systems are:

- *Handle PIDs*: abstract IDs assigned to a resource in accordance to the Handle schema (based on Request for Comment (RFC) 3650^12^); resource owners have to register their resources to a PID service, get the ID and add it to the description of the resource; a PID resolver (included in the system) redirects the end users to the location where the resource resides
- *DOIs (Digital Object Identifiers)*^13^: serial IDs used to uniquely identify digital resources; widely used for electronic documents, such as digitally published journal articles; it is based on the Handle system and it can be accompanied with its own metadata. As with Handle PIDs the resource owner adds the DOI to the description of the resource (30).
- *ORCID (Open Researcher and Contributor ID)*^14^: designed to allow researchers to create a unique ID for themselves which can be attached to publications and resources created by that researcher. This helps to clear up ambiguity when researchers have similar names to others in the field, or when a researcher changes their name (31).

While these identifiers are widely used in the general research domain, there exist identification procedures, of different scale and focus, like the PubMed Identifier (PMID) used for identifying articles in PubMed, or the Maven coordinates used to identify Java libraries. To facilitate search based on identifiers, utilities have been developed to search and match additional identifiers that may have been attached to the same object (article) in other contexts, e.g. find and match additional unique identifiers such as PMID (from PubMed), PMCID (from PMC), Manuscript ID (from a manuscript submission system, e.g. NIHMS, Europe PMC) or DOI (Digital Object Identifier). By using persistent identifiers a biocurator can unambiguously identify and refer to resources of various types, e.g. from publications to domain terminologies and possibly terms themselves, to authors and resource contributors, expecting that he/she will be able to locate such resources even when their initial locations on the web have changed. This property of persistent identification will likewise enable the biocurator or a bioinformatician to reproduce the results of reported experiments conducted by other researchers by appropriately accessing the various types of resources involved in such experiments through resolving (usually by just clicking on) their persistent identifiers.

### Publication repositories

This section describes the online repositories, aggregators and catalogues where publications can be deposited and subsequently discovered. Some have also been presented in ‘Metadata schemata and profiles’ section, from a different angle, i.e. the schema adopted/recommended for the description of resources they host, especially when this is used widely by a variety of resource providers. Scholarly publications can be accessed through a wide range of sites and portals, e.g. publishers' sites, project sites, institutional and thematic repositories, etc. We outline only widespread repositories and aggregators that make available content (or metadata about content) from different sources, mainly open access publications, given that they can function as central points of access. Repositories and aggregators for other types of objects (e.g. language and knowledge resources, language processing tools and services) are presented at the end of each section in this paper, namely, in the ‘Language resources repositories’ and ‘Discovering tools and services’ sections (Table 4).

**Table 4.** A comparison of popular sources for the discovery of and access to publications for TM

Title	Publications	Articles access	Type	Domain
OpenAIRE^a^	14.6 million	Abstracts, some full text articles, reports and project deliverables, open access	Aggregator	Open
Connecting Repositories (CORE)^b^	30.5 million	Abstracts, full text articles, open access	Aggregator	Open
Bielefeld Academic Search Engine (BASE)^c^	91.9 million	Abstracts, full text articles, books and multimedia documents, software and datasets, many open access	Aggregator	Open
PubMed^d^	26 million	Citations, abstracts, no full text articles (in principle)	Aggregator	Biomedical, life sciences
PubMed Central (PMC)^e^	3.9 million	Abstracts and full text of journal articles, open access subset	Repository	Biomedical, life sciences
MEDLINE^f^	22 million	Citations, abstracts	Aggregator	Biomedical, life sciences
Biodiversity Heritage Library^g^	109,382	Abstracts, full text articles, citations, open access	Repository	Biodiversity
arXiv^h^	1.2 million	Full preprints and abstracts	Repository	Biology, physics, computer science, mathematics

We have made a distinction between modes of operation in the ‘Type’ column.

a https://www.openaire.eu/

b https://core.ac.uk/

c https://www.base-search.net/about/en/

d http://www.ncbi.nlm.nih.gov/pubmed

e http://www.ncbi.nlm.nih.gov/pmc/

f https://www.nlm.nih.gov/pubs/factsheets/medline.html

g http://www.biodiversitylibrary.org/

h http://arxiv.org/

While repositories are designated for data depositing, storage and maintenance, aggregators actively harvest data from multiple sources (i.e. repositories) and make them searchable and available in a uniform way. Aggregators can be conceived of as an evolution of hand-coded catalogues. Application profiles and metadata schemata, as discussed in ‘Metadata schemata and profiles’ section, and especially mappings between them to enhance interoperability play a crucial role in the aggregation process and aggregators’ operations.

Based on a pan-European network of institutional, thematic and journal repositories, OpenAIRE (32) brings together and makes accessible a variety of sources including links, publications and research data, improving their discoverability and reusability. Currently, OpenAIRE harvests over 700 data sources that span over 5000 repositories and Open Access journals. Text miners can use OpenAIRE for searching and downloading, where available, publications and/or abstracts of them, and increasingly make use of application programmatic interfaces for querying and mining specific information. In a similar vein, the Knowledge Media Institute of the Open University in the UK has built CORE (Connecting Repositories) aggregating all open access research outputs from repositories and journals worldwide and making them available to the public. CORE harvests openly accessible content available according to the definition of open access. Recently, CORE has started creating data dumps, i.e. big, in the order of hundred thousands, collections of research publications and making available for mining information at different levels. One last example of a publication aggregator is the Bielefeld Academic Search Engine (BASE) (33), which also harvests all kinds of academically relevant material from content sources, normalizes and indexes these data and enables users to search and access the full texts of articles. All three cases of aggregators rely on the widely used OAI-PMH protocol for harvesting publication data.

Specifically focusing on the area of Life Sciences are the repositories of MEDLINE, PubMed and PubMed Central. MEDLINE (34) is the U.S. National Library of Medicine (NLM) bibliographic database, containing >22 million references to journal articles in life sciences with a focus on biomedicine. Records in MEDLINE are indexed with MeSH. PubMed includes >26 million citations for biomedical literature from MEDLINE, life science journals and online books. PubMed citations and abstracts include the fields of biomedicine and health, covering portions of the life sciences, behavioural sciences, chemical sciences and bioengineering. In some cases, citations may include links to full-text articles from PubMed Central and other publisher web sites where the articles were originally published. PubMed Central (PMC), in its turn, is a repository of openly accessible biomedical and life sciences journals literature (35). Scientific publications are deposited by the participating journals and authors of articles that comply with the public access policies of research organizations and funding agencies. Finally, arXiv is a repository of electronic preprints that allows researchers to self-archive and share the full text of their articles before they get published in a journal. It is very popular in the field of physics, but contains documents from several domains, including quantitative biology.

## Knowledge encoding

In the previous section, we covered the problem of acquiring the publications necessary to perform biocuration via TM. However, obtaining the data is not enough—we also need to understand it. A text, especially a scientific publication, is much more than a sequence of words. Some words represent structural elements of a document (headers, chapters, sections and paragraphs) or a sentence (subject, predicate and adjective). Others play special roles, such as URL address, name of a person or citation. Finally, some words or their sequences may be names of concepts that are interesting for a particular purpose. We typically refer to the identification of these special roles as *annotating* and the identified words, with their labels, as *annotations*. These may be obvious for a human reader, but need to be expressed in a strict machine-readable format to allow automatic text processing. The ‘Annotation models’ section describes the most important annotation formats.

During annotation we usually link words or sequences occurring in a text with labels describing their role, e.g. date, title, surname, protein or the concept that they refer to, e.g. a cat, John Smith or *Escherischia coli*, possibly disambiguating between multiple concepts that go by the same name. In both cases, we may refer to existing knowledge resources, e.g. ontologies or dictionaries, as these references allow the annotations to be re-used in future and linked with other similar efforts. This problem is also related to the concept of linked data, which enables semantic search by publishing data in a way that links it with other resources available via the web. However, to create linked data, the target knowledge resource needs to be suitable for referencing, which can be ensured by using one of several suitable interoperable formats. The ‘Formats for knowledge resources’ section enumerates the most popular formats for encoding such resources, while the ‘Useful knowledge resources’ describes exemplar ontologies and vocabularies.

Creating an annotated corpus or knowledge resource, in particular when done manually, is a time consuming process. The products of such efforts are sometimes used for many years, but they also may become inaccessible if an under-specified or poorly documented format has been employed. Furthermore, a lot can be gained by comparing or aggregating annotations from different corpora, which is only doable if the semantics of annotations across corpora are consistent. How can we make our research reusable and permanent? We need to take care of the interoperability of every aspect of our work—protocols, formats, vocabularies, knowledge bases, etc. Annotation is a great example. If we use an interoperable standardized annotation format and refer to publicly available, well established knowledge resources, everyone will benefit.

### Annotation models

Annotation is the process of adding supplemental information to a text in natural language. The annotations are produced as an output of an automatic or manual tool and may be:

- treated as an input for further automatic stages,
- visualized for interpretation by humans,
- stored as a corpus.

In each of these cases, we need the annotations to follow a precisely defined data format. This section is devoted to presenting such formats, both domain-dependent and generic. Table 5 summarizes the different formats which are commonly used.

**Table 5.** A comparison of annotation formats used in TM

Model	Domain	Serialization formats	API	Type
BioC^a^	Biomedical	XML	Reference APIs in multiple languages	Stand-off
BioNLP shared task TSV^b^	Biomedical	TSV	No	Stand-off
BRAT format^c^	Generic	TSV	No	Stand-off
Pubtator^d^	Biomedical	TSV	No	Stand-off
TEI^e^	Generic	XML	Via XSLT^f^	Stand-off
NIF^g^	Generic	RDF	No	Stand-off
LIF^h^	Generic	RDF	Reference API in Java^i^	Stand-off
IOB	Generic	TSV	Third-party APIs in several languages	In-line
Open Annotation^j^	Generic	RDF	No	Stand-off
CAS (UIMA)^k^	Generic	XML (XMI)	Reference APIs in Java and C ++^l^	Stand-off and in-line
GATE annotation format^m^	Generic	Several	Reference API in Java^n^	Stand-off and in-line
LAF/GrAF^o^	Generic	XML	No	Stand-off
PubAnnotation^p^	Generic	JSON	REST API to annotation store^q^	Stand-off

‘API’ stands for application programming interface and refers to whether there is a suitable library for use with this format. The domain column denotes the typical category of information encoded with this format.

a http://bioc.sourceforge.net/

b http://2011.bionlp-st.org/home/file-formats

c http://brat.nlplab.org/standoff.html

d http://www.ncbi.nlm.nih.gov/CBBresearch/Lu/Demo/PubTator/

e http://www.tei-c.org/Guidelines/P5/

f http://www.tei-c.org/Tools/Stylesheets/

g http://persistence.uni-leipzig.org/nlp2rdf/

h http://wiki.lappsgrid.org/interchange/

i http://mvnrepository.com/artifact/org.lappsgrid/vocabulary

j http://www.w3.org/ns/oa

k https://uima.apache.org/d/uimaj-2.7.0/references.html#ugr.ref.cas

l https://uima.apache.org/downloads/releaseDocs/2.1.0-incubating/docs/html/tutorials_and_users_guides/tutorials_and_users_guides.html

m https://gate.ac.uk/sale/tao/splitch5.html

n http://jenkins.gate.ac.uk/job/GATE-Nightly/javadoc/

o ISO 24612:2012 – http://www.iso.org/iso/catalogue_detail.htm?csnumber=37326

p http://www.pubannotation.org/docs/annotation-format/

q http://www.pubannotation.org/docs/api/

All of the formats presented in Table 5 have a data model that allows the representation of annotations independently from the domain application. The Domain column indicates the salient communities that use the format. Generic formats are used in very diverse domains, including the biomedical. Even though they are generic in their design, the format with the domain ‘biomedical’ is mainly used within the biomedical text mining and natural language processing communities.

In order to grasp the specificities of each format and to be able to choose one, it is important to recall the goals that motivated the specification of each format. We can classify these objectives in three broad categories, as outlined below.

#### Formal representation and sharing

The goal is to provide a formal representation framework for linguistic and semantic annotations of texts. The objectives are to normalize the representation of annotations inside the community of annotation producers, to allow the exposure of annotations to peers. Some of these models were designed by a committee of language annotation professionals, who attempts to cover the widest range of annotation situations in order to build a complete and expressive format. LAF (36), XMI^15^, and Open Annotation (37) are examples of models designed with this goal in mind. These formats are suited to expose and share annotations, especially if these annotations are complex. Normative formats also have the advantage of being known and recognized by a large number of tools and services, although the user should always take care to ensure that the format they choose is suitable for their purpose and has sufficient tool support to be useful.

#### Operational interoperability

Some formats presented in Table 5, such as NLP Interchange Format (NIF) (38), were specifically designed to be flexible and generic such that they can be used as interchange formats in arbitrary analysis workflows. Workflows play an important role in TM and NLP because the operational results are rarely produced by a single piece of software or method. Useful outputs require an accumulation of coordinated process steps. For instance, most applications would need sentence splitting, tokenization, POS-tagging, several steps of named-entity recognition and so on. More complex applications may also need syntactic parsing, relation extraction, semantic labelling and more. Each step is designed to achieve a specific and atomic task and operates on the text as well as the output of previous steps.

In the NLP community, workflow implementations commonly wrap individual tools in order to have uniform access to their input, output and parameters. In this case, the workflow works on a single annotation model called the ‘pivot model’. The output of each component tool is translated into the pivot model, conversely the annotations expressed in the pivot model are translated into the native tool input format. Performance is one of the main design principles of these formats. BioC (39), GATE annotation format, LIF (40) and CAS (41) are formats designed to be processed by BioC, GATE (42), LAPPS (43) and UIMA (8) workflows, respectively.

These formats present the advantage of giving direct access to the processing tools available for the respective workflow engine. Although annotations can be shared, they usually confine the annotations to the ecosystem of the processing tools of the workflow engine.

Other formats have been designed for a more specific use, such as storing outputs of manual annotation such as BRAT (44), or encoding corpora e.g. TEI (18).

#### Human–machine readability

Other formats are designed to be, at the same time, processed by machines and read by humans. Indeed NLP and Information Extraction developers need annotations in formats that they can use with their tools, especially machine learning tools, but also that they can read in order to grasp the data and analyse the errors of tools in development and production.

Most of these formats have been designed as data formats supported by NLP and IE challenges: BioNLP Shared Task (BioNLP-ST TSV) (45), BioCreative (PubTator) (46) and CoNLL (TSV/IOB). Challenges are important events that gather the NLP and IE community. They allow the assessment of the performance of tools and methods against real-life data. Typically, challenge participants will feed annotations to automatic tools, as well as look into annotations.

The main advantage in exposing one’s own annotations in these formats is that they can be processed by the most state-of-the-art research software.

#### Format paradigm

The annotations may be inserted in the text (in-line, similar to tags in HTML) or provided as a separate file (stand-off). The overwhelming majority of formats opt for stand-off annotations. On one hand in-line annotations have serious limitations for representing complex structures like overlapping spans, discontinuous phrases, or relations. On the other hand stand-off formats allow the transmission of annotations separately from the annotated text that cannot always be distributed for legal reasons.

There is a strong tension between human readability and genericity of a format. The more complex the structures to be encoded become, the more identifiers and cross-references need to be introduced which gradually erodes human readability. For example, the CONLL 2006 format is a fixed scheme format with a good human readability; the TSV format used by WebAnno (47) is a variable-scheme format that tries to strike a compromise here by scaling the encoding complexity. The simpler the annotations are, the more human-readable is the format, for example see PubAnnotation (48). Cross-references and identifiers are introduced on a by-need basis, not preemptively; the GrAF XML format is a variable-scheme format using references and identifiers a lot and is hardly human-readable even for documents with only simple annotations

In fact, in-line annotations have two advantages in rather niche situations. In-line annotations map directly to mark-up formats used natively by several visualization tools. In general, in-line formats are more easily read by humans. Also they are particularly well suited as input data for algorithms widely used in named-entity recognition, like Hidden Markov Models (HMMs) or Conditional Random Fields (CRFs). The transformation from in-line to stand-off is trivial as stand-off annotations are more expressive. Transforming back to in-line from stand-off can be difficult, especially if the annotation has passed the boundaries of the expressivity of the in-line annotation format. In-line formats may be chosen as they are easier for a human to read, however in the general case we recommend stand-off formats.

### Formats for knowledge resources

In this and the next section, we focus on a special type of resources that play the role of knowledge sources. This may be purely linguistic information (e.g. morphology dictionaries), general world knowledge (e.g. open-domain ontologies) or very specific use (e.g. translation) or domain knowledge. First, we describe the formats used to encode such resources. Table 6 contains a list of them with the most basic features.

**Table 6.** A comparison of formats for the encoding of different types of knowledge resources

Format	Resource type	Serialization	Libraries available
TMF/TBX^a^	Terminologies	XML	Yes^b^
LMF^c^	Lexica	LMF	No
SKOS^d^	Thesauri	RDF	Yes (RDF)^e^
OWL^f^	Ontologies	several	Yes^g^
OBO^h^	Ontologies	own	Yes^i^
Ontolex^j^	Lexica relative to ontologies	RDF	Yes^k^
TMX^l^	Translation memories	XML	Yes^m^
XLIFF^n^	Translation memories	XML	Yes^o^

‘Libraries available’ refers to whether there is a suitable library for use with this format.

a http://www.tbxinfo.net/

b http://www.tbxinfo.net/tbx-downloads/

c http://www.lexicalmarkupframework.org/

d https://www.w3.org/TR/skos-reference/

e https://www.w3.org/2004/02/skos/tools

f https://www.w3.org/OWL/

g http://owlapi.sourceforge.net/

h ftp://ftp.geneontology.org/pub/go/www/GO.format.obo-1_4.shtml

i http://oboedit.org/?page=javadocs

j https://www.w3.org/community/ontolex/wiki/Final_Model_Specification

k https://github.com/cimiano/ontolex/blob/master/Ontologies/ontolex.owl

l http://xml.coverpages.org/tmxSpec971212.html

m http://docs.transifex.com/api/tm/

n http://docs.oasis-open.org/xliff/xliff-core/xliff-core.html

o http://www.opentag.com/xliff.htm#Resources

A variety of formats is necessary to represent the organization and actual content of the linguistic and conceptual resources that are used to feed TM and NLP software. Their adoption by resource developers can be explained as follows.

#### The nature of content elements

The formats listed in Table 6 correspond more or less to families of resources that allow the exploitation by software of different facets of knowledge ranging from words to concepts.

Lexica provide descriptions of lexemes, i.e. a language's words, focusing on morphology, syntax and sometimes semantics, all of which are elements precisely described by the Lexical Markup Framework (LMF) (49). In this work, vocabularies are to be understood as sets of elements of knowledge, possibly structured and controlled. Their typical function is to represent consensual meaning of concepts inside domain communities. Vocabularies cover gazetteers, authority lists, thesauri, terminologies, classification schemes, etc. TMF/TBX and SKOS are particularly suited for this. OWL and OBO (50), initially developed for the ontological representation of concepts, are also commonly used for implementing borderline vocabularies also called termino-ontologies. Finally, translation memories record pairs of segments of texts that have previously been translated. Both TMX and XLIFF like the majority of translation memory standards focus on the context rather than on the internal structure of the segments. Note that all formats listed support multilinguality.

Considering the heterogeneity of the nature of contents represented in resources, most of the formats listed in Table 6 are not exchangeable in a TM workflow. Indeed, exchangeability is generally neither possible nor useful as each type of component, e.g. word disambiguation, consumes one specific or a given set of resource types, e.g. lexica or vocabularies. Only inside the same level of linguistic or knowledge representation are the formats exchangeable such as between OBO and OWL, for which translation routines already exist.

#### Fitness towards TM

It is worth noticing that LMF is the only format from the list above that was designed in the context of ISO/TC37 specifically to feed into the NLP process. UBY-LMF is an example of instantiation of the LMF model. It has been used in TM pipelines on, for instance, word sense disambiguation or text classification.

Other formats like OWL, SKOS and, to a lesser extent, OBO are central, especially since the emergence of the Semantic Web, as they are widely adopted by domain specific communities. More and more pointed and high value knowledge resources are thus made available for TM advanced tasks in addition to already widespread general knowledge bases. However, OWL, OBO and SKOS resources often lack information on the lexical level because the formats are not suited and such resources are created with information organization or reasoning purposes. They may need reworking before exploitation in a TM pipeline. Ontolex, the core of the lexicon model for ontologies (LEMON), was created to overcome these weaknesses of OWL.

#### Interoperability

The issue of the interoperability of knowledge resources has to be considered according to two aspects.

First, resources have to interact with TM pipelines both as inputs and outputs of specific components. The formats listed in Table 6 may not be sufficient to qualify the compliance of a resource with a tool. The user generally needs to read the resource’s documentation along with the software’s documentation. In the best case scenario, these sources of documentation specify the appropriate and necessary elements for a given task, however there is no guarantee that the documentation will be clearly written. This hinders the design of tailor-made TM workflows by non-specialists as they require an in-depth knowledge of the resources and tools.

In addition, knowledge resources need to be interoperable with each other. Users may want to merge existing resources to answer their specific needs. This reduces development costs and allows them to benefit from expertize they probably do not have, particularly on specialized domains. Most of the formats above offer mechanisms towards this kind of interoperability thanks to available libraries.

Yet, there are still many knowledge resources that are not using standards because for instance they are tied in to a given software, or felt not to be necessary by the developer. The adoption of standards for resources is an essential driver towards flexible and reusable TM pipelines.

### Useful knowledge resources

Although it is not possible to enumerate all knowledge sources used for TM and biocuration, we try to outline several examples here, focusing on their interoperability. We have focused on resources from the life sciences domain, but at the end of this section the reader will find some more general examples with an explanation of their role in text mining for biocuration. Table 7 describes the resources we have highlighted, including their most important features.

**Table 7.** A comparison of popular knowledge resources, typically used in TM for the life sciences

Name	Type	Domain	Size	Format	License
Uniprot^a^	Knowledge base	Proteomics	63 million sequences	Own, RDF, FASTA	CC
UMLS^b^	Thesaurus	Biomedical	3.2 million concepts	Own	Proprietary
Gene Ontology^c^	Ontology	Genetics	44 000 terms	OBO	CC
Agrovoc^d^	Thesaurus	Agriculture	32 000 concepts	RDF	CC
HPO^e^	Vocabulary	Human phenotype	10 000 terms	OBO, OWL, RDF	Free to use
CNO^f^	Vocabulary	Neuroscience	395 classes	OWL, RDF	CC
CARO^g^	Ontology	Anatomy	96 classes	OBO, OWL	Unspecified

These resources differ in terms of type, domain and intended use. These differences make size difficult to compare as different resources have different base elements. Nonetheless, we have presented the table in an approximate order of size from largest to smallest.

a http://www.uniprot.org/

b https://www.nlm.nih.gov/research/umls/

c http://geneontology.org/

d http://aims.fao.org/vest-registry/vocabularies/agrovoc-multilingual-agricultural-thesaurus

e http://human-phenotype-ontology.github.io/

f https://bioportal.bioontology.org/ontologies/CNO

g http://www.obofoundry.org/ontology/caro.html

#### Domain specific resources

Domain specific resources capture the formal knowledge or the language used in a delimited scientific or technical domain. Domain specific resources are built by domain experts, so the entries are usually familiar to biocurators. These resources are used for labelling document topics, or to extract occurrences of entries in the content. The automatic processing of documents links documents to entries in domain specific resources, and thus helps biocurators in a systematic approach to their task.

The majority of domain specific resources mentioned in Table 7 are expressed in OWL, or OBO. Resources in OBO can be easily translated into OWL/RDF, since the OBO model is a subset of OWL. The reverse is usually possible, at least partially. UMLS (51) uses a specific format because it is an aggregation of very diverse nomenclatures, some of them originating from before the development of OBO and OWL. Uniprot (52) is a curated resource containing protein sequences and information on their functions, it has used a specific format since its conception in 1986. There are two approaches for domain specific applications that require these resources. Either they understand natively the formats, or they reformulate them into OWL/RDF. For some resources, e.g. Agrovoc (53), RDF is the only available format.

One of the main interoperability challenges for domain specific resources is that they do not always cover distinct subdomains. Therefore, different resources contain common objects, however they do not necessarily carry the same name and identifier. For instance both the UMLS and CARO (54) contain the concept ‘basal lamina’. An application that uses overlapping resources has two courses of action. They can act as if the resources were distinct, at the risk of duplicating information. Or they can map objects from different resources, which may prove difficult and costly. In the best case, resources already contain cross-references to objects in other resources, but cross-references are not typed and could mean object equivalence as well as just ‘see-also’ relationships. Sometimes term definitions may as well contain concepts from other ontologies and link to them, e.g. HPO (55). Ontology alignment and terminology are whole domains of research that aim to produce such mappings automatically using the objects labels or the comparison of the structures of resources—for biomedical examples, see Bio2RDF (56) and KaBOB (57).

When adopting a TM system in order to assist their task, biocurators face the difficulty to choose among resources. The choice must be driven by three main criteria: the topic coverage, the quality of the resource and licensing.

The topic coverage is the most obvious criterion: the resource must address the domain or subdomain at hand. The main reason a resource becomes popular is that it covers an exclusive topic, and it is well documented so the coverage is made well known. Even though several resources may seem to compete in a specific topic, they may adopt different points of view or address different levels of granularity. We advise that biocurators investigate the documentation in order to understand the precise boundaries and the point of view of the considered resources.

The quality of knowledge resources is difficult to define and assess. Unfortunately, the most reliable way to assess the quality of a resource is experimenting with services using this resource. However, some properties can be checked beforehand to ensure that the service that integrates the resource will meet one’s expectations. Licensing is also key, as a biocurator must select resources which will be compatible with their final intended use. For example, some resources may come attached with a non-commercial licence which may not be suitable in an industrial setting.

##### Development process and curation

The process of development and maintenance of a resource is a good indicator of its quality. Resources under active development, supported either by a recognized institution, or by a stable committee are likely to be reliable. Quality resources also have to be curated, thus a clear and sensible policy for accepting contributions indicates a coherent resource. In the best case, the methodology of construction is described in a scientific publication.

Almost all the resources mentioned in Table 7 are manually curated, which means that they are the result of a process involving humans reading relevant publications or other knowledge sources and extracting necessary information. The only exception is Uniprot, which includes a section (UniProtKB/TrEMBL) of automatically annotated and classified entries. Automatic and semi-automatic solutions permit a biocurator to increase the coverage with reduced human effort, but also result in lower annotation quality because of limited accuracy of automatic methods. In the general domain (as shown below), most of the knowledge resources that we have covered are automatically extracted from textual databases, particularly from Wikipedia.

##### Community of users

A widely popular resource might be a good one, however one has to check if the resources are used with the same objectives. For instance Gene Ontology (58) is extremely popular, however it was designed to normalize functional annotations of genes. This resource has drawn a remarkable attention from the TM and NLP community. However on close examination, the extraction of GO concepts from text content is still a research subject since it has proven to be challenging (59).

##### Semantic strictness

Ontologies and knowledge bases contain intensional knowledge that will be used by TM tools for inferences. If a service uses inappropriate inferences on a resource, or if a resource contains approximative intensional knowledge, then the impact on the output can be dramatic. For instance, one can check if ‘is-a’ relationships in an ontology actually denote strict specialization, and not related or weaker relations. For instance, a tool may take advantage of the taxonomic structure of a thesaurus in order to improve the extraction or the retrieval of information in the text. However, this tool can propagate errors if the terms are misplaced or inappropriate to the context at hand.

##### Lexicalization

Lexicalization is the property of a resource to capture the majority of the specific language associated with the domain specific concepts. A good lexicalization will ensure that information can be properly and comprehensively extracted from the text’s content. For instance, one can check if the most common synonyms and acronyms are present in the resource, or conversely if ambiguous terms carry enough context within the resource to allow for automatic disambiguation.

#### General domain and linguistic resources

Resources that are not domain-specific and linguistic resources are often used in TM tools to complement domain-specific resources. Indeed, linguistic resources are helpful, as domain-specific resources seldom capture the whole diversity of language used to express the objects they contain. Some domain-specific resources contain synonyms, but it is impossible to comprehend all the typographic, morphological and syntactic variations. This knowledge is nonetheless very important for the detection of entities in the text of the documents. Without this knowledge, the TM tools may miss mentions of concepts, or be confused by ambiguous mentions or concepts that have similar lexical manifestations. Princeton WordNet (60), OliA (61) and GOLD (62) are among the most widely used linguistic resources.

All the information needed by biocurators is not necessarily domain-specific, for instance TM tools can extract and present general-domain entities, like persons, countries, or organizations, in order to assist them. Resources derived from Wikipedia are often used to this effect: Wikidata (63), DBpedia (64), Freebase (65) and YAGO (66).

### Language resources repositories

It is important for the researcher to know where to look for resources. In the table below, we have listed repositories which are useful for TM in the life sciences. These repositories allow a user to browse for content, search for relevant resources, download resources (often for free) and upload their own resources for others to discover and use. Resources will typically be in the formats suggested in the ‘‘Formats for knowledge resources’ section . The format of the resource will usually be included as part of the metadata in the repositories to help the researcher decide if the resource will be suitable for their needs.

The repositories listed in Table 8 allow the visitor to identify and localize the resources that will answer his needs. Such repositories that are available on the web can be divided into three kinds: catalogues, directories and metadata repositories. Their relation to metadata and the services they offer will be discussed hereafter.

**Table 8.** Repositories for the curation of language resources, indexing language resources that are useful for the general domain and the life sciences

Title	Available records	Type of content	Accessibility (Download)	Accessibility (Upload)	Domain
ELRA Catalogue of Language Resources^a^	1137	Corpora, lexica	Some Paid	Restricted	Language technology
LDC catalogue^b^	Over 900 resources	Corpora	Some Paid	Restricted	Language technology
VEST Registry^c^	118	Vocabularies, standards, tools	Open	Registration upon request	agriculture, food, environment
AgroPortal^d^	35	Vocabularies	Open	Registration upon request	agriculture, environment
BioPortal^e^	576	Vocabularies	Open	Registration upon request	biology, health
CLARIN VLO^f^	876 743 records	Various	Open	Upon request	Language technology
META-SHARE^g^	More than 2700	Corpora, lexica, language descriptions, tools/services	Open	Registration upon request	Language technology
Stav corpora^h^	30	Annotated corpora	Open	Closed	biomedical

a http://catalog.elra.info/

b https://catalog.ldc.upenn.edu/

c http://aims.fao.org/vest-registry

d http://agroportal.lirmm.fr/

e http://bioportal.bioontology.org/

f https://www.clarin.eu/content/virtual-language-observatory

g http://metashare.elda.org/

h http://corpora.informatik.hu-berlin.de/

#### Different types of store

##### Catalogues

The two catalogues above, namely ELRA (67) and LDC, meet the Longman definition of catalogue as ‘a complete list of things that you can look at, buy, or use, for example in a library or at an art show’^16^. The operators managing those catalogues play the role of brokers carefully selecting the resources and their suppliers who state the conditions of distribution (license, possibly price). The user is a member and accesses the resources on conditions depending on his status (academic/private or profit/non-profit) and sometimes the use he wants to make of the resource (research or commercial). Resources are generally high-valued ones. Samples, when proposed, allow the user to evaluate the resource towards the targeted task before paying.

##### Directories

The Vest Registry and CLARIN VLO (19) are directories as they simply expose information on a selection of resources. They provide the necessary information for the user to discover the resources, generally through a web link (usually a persistent identifier like the ones mentioned in the ‘Introduction’ section) to the resource or its original web page. Contributors to the VEST Registry are a small community of registered users who notify valuable resources for the agriculture community. CLARIN VLO harvests and presents metadata from many providers from a variety of European countries.

##### Metadata repositories

META-SHARE (20), BioPortal (68) and AgroPortal (69) store and make both metadata and their associated resources directly available to the user for download. While META-SHARE has a general thematic scope, BioPortal and AgroPortal are respectively dedicated to Biomedicine and Agriculture. Furthermore, the META-SHARE portal features an aggregator, enabling in fact a federated access to a great number of repositories organized as nodes in a network. These reasons explain the sensible difference in size between them, AgroPortal having, in addition, been launched only a few months before the time this paper was written.

The Stav repository (70) differs from other repositories of biomedical corpora in the way that it presents documents to a user. Instead of downloading an annotated file, one can visualize the annotation in an on-line tool. Available corpora, although not numerous, cover a wide range of annotation types, from a variety of named entities to complex events.

#### The importance of metadata

In such repositories, especially large ones, poor metadata leads to the user looking for a needle in a haystack. In this respect, setting up the metadata schema that underpins either a catalogue, a directory or a metadata repository is a crucial step in the whole system design process. Too few means less services to the final user, too many or too complex may lead to providers not being able or not willing to provide the information relating to their resources. Metadata have functions translated into functionalities or services in the repositories.

##### Discovery

Visitors use sets of metadata as relevant criteria to discriminate one or some resources among all. These include descriptive (e.g. type, language, domain, curatorial information), technical (e.g. format, tool compatibility, creation process) and usage metadata (e.g. license, popularity). This information is generally materialized on a repository’s home pages as drop-down menus and further accessible through facets during the search process.

META-SHARE proposes no < 19 criteria to filter out resources. In repositories and directories collecting data from various sources, a key challenge is the mapping of original metadata fields into a common meaningful schema. The growing uptake of standard metadata schemata by both resource producers and stores combined with the achievements of international initiatives and infrastructure projects like META-SHARE or CLARIN make the mapping work easier. However, value lists associated to some metadata fields are still stumbling blocks. While lists for languages or countries are widely shared, building consensus on subjects, resource types, media types, or formats is still work in progress, through initiatives like COAR.

##### Identification and localization

Having a multiplicity of access points to knowledge resources is a necessity to serve TM stakeholders with different cultures and habits. But this leads to a situation where resources are duplicated many times with the risk of creating inconsistencies from one repository to another. In order to be reproducible, TM and NLP processes need to refer explicitly to resources and the specific versions they use. Elements of different metadata schemata, like the persistent identifiers mentioned in ‘Mechanisms used for the identification of resources’ section, enable such referencing. Using persistent identifiers for language resources has only recently been established, and the most widely used identification system is Handle PIDs. Still, generic ones like the DOIs (see ‘Mechanisms used for the identification of resources’ section) which allow the identification of both resources and their versions, are also used by some resource providers and/or distributors. Resource developers should be encouraged to use persistent identifiers in combination with relevant metadata elements when publishing.

The sustainable hosting of resources is also a concern, in particular for repositories that reference distant content, as too many broken links are a reason for the user to give up a directory. This sustainability in hosting and, in a linked manner, in accessing a resource is also key to ensure its reuse and popularity. Common repositories can offer this service while other hosting solutions like simple web pages generally do not.

#### Value added services

Some repositories and particularly small domain specific repositories offer more than just the possibilities to discover and download resources. BioPortal and AgroPortal propose an integrated environment for browsing, searching, sharing and commenting on resources. Advanced functionalities allow the user to simply evaluate the adequacy of one or several resources towards a given text. More interesting is the possibility to compute and store mappings between concepts, thus creating a conceptual network across resources. Such mappings are valuable for NLP related tasks like annotation, resource building or word disambiguation.

The issues addressed so far have only concerned human users. Leaving aside catalogues that address only people’s needs, almost all recent stores also provide services to machines through Application Programming Interfaces (APIs). In addition, Web Semantic technologies, SPARQL in particular, increase the potential of communication between processes and repositories. In that perspective, standards for metadata and resource formats are even more crucial in allowing programs to select, identify and access resources from repositories in an unambiguous and constant manner.

## Tools and services

The needs of a text miner vary from task to task. In the best case scenario, another researcher will have already created a tool or web service that can be reused for another purpose. At the end of this section, we have listed several useful resources that can be used to discover tools and services for TM. If the text miner cannot find a pre-existing tool then they must look to develop their own. However, not all tools need to be programmed from scratch. Some can be created simply by taking multiple existing tools and reengineering them to jointly act as a new tool. For such a task, workflow systems may be useful for both the novice and expert alike. A typical workflow management system (WMS) provides a collection of reusable components and the ability to link the processing of these together in an intelligible manner. The WMS typically consists of the following major blocks:

- a workflow description language;
- a workflow engine that interprets the workflow description language and runs the workflow;
- a collection of components from which workflows may be assembled;
- a repository where components and workflows are stored and may be shared with other users;
- possibly a workbench which allows a user to graphically access the repository, compose components into workflows and run these using the engine.

In particular, the ability to compose workflows by using other workflows as components makes such systems very flexible and powerful.

Most of the software packages examined here do not support all aspects of a WMS. Based on which aspects are supported, we apply a fine-grained categorization: the software packages mentioned in this section cover five categories with most packages belonging to more than one categories:

1. *Interoperability frameworks*: provide a data exchange model, a component model for analytics components, a workflow model for creating pipelines, and a workflow engine for executing pipelines;
2. *Component collections*: collections of components based on an interoperability framework, including analytics components, but also input/output converters;
3. *Type systems*: a data interchange vocabulary that enables the interoperability between components, typically within a single component collection;
4. *Analytics tools*: standalone natural language processing tools that are typically not interoperable with one another and thus wrapped for use within a specific interoperability framework;
5. *Workbenches*: user-oriented tools with a graphical user interface or web interface by which the user can build and execute analytics pipelines.

In some cases, identifying which part of the software relates to which of the above categories is difficult. For example, in Stanford CoreNLP, which does offer a type system and a workflow engine, the separation is not as clearly reflected in the system architecture and advertized separately as in UIMA or GATE.

In the course of the present section, we will present a variety of WMSes that can be used to help the researcher in TM. In the ‘Text mining workflow management systems’ section, we show WMSes which are designed specifically for the purpose of TM. ‘General purpose workflow engines’ section presents a further list of WMSes which are designed for general purpose research. It is possible to use these for TM and this could be appropriate for a researcher who has previous experience in one of these platforms. Finally, ‘Discovering tools and services’ section presents repositories that are useful for the discovery and storage of TM services.

### Text mining workflow management systems

Almost any TM application is formulated as a workflow of operational modules. Each component performs a specific analysis step on the text and when it is finished, the next component begins. Some components may be generic and can be used for many different applications (file lookup, sentence splitting, parsing, entity identification, relation extraction, etc.), whereas other components may be less common, or even built specifically for the task at hand. A TM WMS defines a common structure for these components and facilitates the creation of a workflow of existing components as well as helping with the integration of new components. The variety of functionality that software packages in this area provide is rich—often packages provide more than one functionality. To make this approachable, we organize the software packages into four major categories, based on what we perceive to be the predominant functionality of a package:

- *Processing frameworks*: software frameworks which focus around one specific data model and component model (Table 9).
- *Analytics packages*: Software libraries that provide NLP/TDM related analytics (Table 10).
- *Component collections*: Software packages that integrate analytics packages with a processing framework (Table 11).
- *Analytics workbenches*: User-facing tools which permit the composition of components into workflows, the execution of workflows, and the inspection of results(Table 12).

**Table 9.** A comparison of popular interoperability frameworks and supported workflows

Name	Workflow description language	Workflow engine	Programming language	License
Alvis^a^	Alvis	Alvis	Java	ALv2
Apache UIMA^b^	Aggregates	Aggregates	Java/C ++	ALv2
	CPE	CPE		
	UIMA AS	UIMA AS		
	RUTA	RUTA		
	UIMA DUCC	UIMA DUCC		
GATE Embedded^c^	GATE Applications	GATE Embedded	Java	LGPL
Heart of Gold^d^	Yes (unnamed)	MoCoMan	Java/Python	LGPL

a http://www.quaero.org/module_technologique/alvis-nlp-alvis-natural-language-processing/

b https://uima.apache.org/

c https://gate.ac.uk/family/embedded.html

d http://heartofgold.dfki.de/

**Table 10.** A comparison of popular analytics packages

Name	Native processing framework support	Programming language	Repository	License
Apache OpenNLP^a^	UIMA	Java	Maven	ALv2
NLP4J (aka Emory NLP)^b^	No	Java	Maven	ALv2
FreeLing^c^ (73)	No	C ++	No	AGPL + commercial
NLTK^d^ (74)	No	Python	PyPI	ALv2
LingPipe^e^	No	Java	Maven	AGPL + commercial
Stanford CoreNLP^f^ (75)	No	Java	Maven	GPL + commercial

a https://opennlp.apache.org/

b https://github.com/emorynlp/nlp4j

c http://nlp.lsi.upc.edu/freeling/

d http://www.nltk.org/

e http://alias-i.com/lingpipe/

f http://stanfordnlp.github.io/CoreNLP/

**Table 11.** A comparison of popular component collections

Name	Focus area	Processing framework	Repository	Programming language	License
Apache cTAKES^a^	Medical records	UIMA	Maven	Java	ALv2
Bluima^b^	Biomedical	UIMA	Maven	Java	ALv2
ClearTK^c^	Machine Learning	UIMA	Maven	Java	BSD/GPL
DKPro Core^d^	Linguistic analysis	UIMA	Maven	Java	ALv2/GPL
JCoRe^e^	Biomedical	UIMA	Maven	Java	LGPL/GPL
BioNLP-UIMA^f^	Biomedical	UIMA	Maven	Java	BSD
GATE built-in component collection^g^	Linguistic analysis and information extraction	GATE	GATE	Java	LGPL/GPL
NaCTeM collection^h^ Biomedical	UIMA	None	Java	Proprietary	
Semantic Software Lab collection^i^	Biomedical	GATE	GATE	Java	LGPL/GPL

a http://ctakes.apache.org/

b https://github.com/BlueBrain/bluima

c https://cleartk.github.io/cleartk/

d https://dkpro.github.io/dkpro-core/

e http://julielab.github.io/

f http://bionlp.sourceforge.net/

g https://gate.ac.uk/

h http://argo.nactem.ac.uk/

i http://www.semanticsoftware.info

**Table 12.** A comparison of popular analytics workbenches

Name	Processing framework	UI	Component collection	External repositories	License
Argo^a^	UIMA	Web-based (service)	NaCTeM	No	Proprietary
CLARIN-D WebLicht^b^	Proprietary	Web-based (service)	Built-in	No	Proprietary
GATE Developer^c^	GATE	Installable application	Built-in External	GATE Repositories	LGPL
U-Compare^d^	UIMA	Installable application	Built-in	no	Proprietary
UIMA Ruta^e^	UIMA	Installable application (Eclipse plugin)	UIMA-based (e.g. DKPro Core, …)	Yes (via Maven)	ALv2
LAPPS Grid Galaxy^f^	UIMA + GATE via Galaxy	Web-based, installable application	Multiple (e.g. GATE, DKPro Core, …)	Galaxy tool shack	ALv2

a http://argo.nactem.ac.uk/

b http://www.clarin-d.de/en/language-resources-and-services/weblicht

c https://gate.ac.uk/family/developer.html

d http://nactem.ac.uk/ucompare/

e https://uima.apache.org/ruta.html

f http://galaxy.lappsgrid.org/

It is also notable that most of the software is implemented in Java. The Java platform provides interoperability across most hardware and operating system platforms (e.g. Windows, Linux, OS X). It also facilitates interoperability between the different software packages. For example, Java-based component collections can more easily integrate other Java-based software than software implemented in C/C ++ or Python (although this is not impossible).

#### Processing frameworks

In terms of processing frameworks, the Apache UIMA framework and the GATE framework (42) appear to be the strongest and more widely used in the TM community than Alvis (71) or Heart of Gold (72).

Several of the component collections presented below are UIMA-based and in principle interoperable at the level of workflows and data model. In particular, we can infer that the expressiveness and flexibility of the UIMA data model appears to fulfil the needs of the community. However, each of the UIMA-based software packages uses its own specific annotation type system. This means that things that are conceptually the same, e.g. tokens or sentences, have different names and often different properties and relations to each other. Consequently, the practical interoperability here is limited.

#### Analytics packages

The list given here is by no means exhaustive, but it is rather representative of software packages that support a whole set of analysis tasks (tokenising, POS tagging, parsing, etc.) instead of only a single task.

Most of the analytics software presented here is in principle language-independent and only specialized to a particular language or domain by models, e.g. machine learning classifiers trained for a specific language or domain, rules created for the extraction of specific information, domain-specific vocabularies and knowledge resources, etc. However, the level of support across languages varies dramatically. Models and resources for English are available in almost all software packages, further well-supported languages include German, French, Spanish, Chinese and Arabic, followed by a long tail of limited support for other languages.

#### Component collections

Component collections represent a piece of software that sits in between a processing framework and an analytics tool. The software acts as an adapter that allows combining analytics tools coming from different software packages and created by different providers into workflows. Often, component collections wrap third-party tools that are also separately available as software packages, but occasionally analytics are provided only in the form of a component for a specific framework.

Component collections typically focus on a particular area of language analysis and are centered around an annotation scheme which models this area in particular. Some collections are focused on a very specific use-case, e.g. cTAKES (76) on the analysis of clinical text, Bluima (77) on the extraction of neuroscientific content and ClearTK (78) on adding machine learning functionality to UIMA. Others host different tools for the general domain of life sciences, e.g. JcoRe (79), the NaCTeM (National Centre for Text Mining) collection (9), BioNLP UIMA (80) and the Semantic Software Lab collection. The third category, including collections like DKPro Core or ClearTK, provide a broad range of rather low-level analytics tools that act as a toolkit for the implementation of many different kinds of use-cases.

Giving a clear indication of the size of a component collection is difficult. For example, if one component can be parametrized with three different models for three different languages, should it be counted three times or only once? Some component collections offer separate wrappers for individual functions of a tool, e.g. for a tool that combines part-of-speech tagging and lemmatizing. Other collections only offer a single wrapper offering both functionalities. Numbers found on the websites of the respective software packages use different counting strategies for components and are therefore incomparable.

The licenses stated for the component collections refer to the primary license of the wrapper code. The actually wrapped third-party software packages often have other licenses. Also, specific components in a collection may have other licenses, e.g. due to GPL copyleft provisions.

#### Workbenches

Using analytics software or components programmatically in the sense of a software library requires programming skills. This is a major problem for the larger adoption of NLP/TDM technologies in less programming-oriented domains. Workbenches aim to facilitate the use of analytics components by providing a graphical user interface that allows a user to browse components, assemble them into workflows, execute these workflows, and inspect the results.

The workbenches listed here were created with a particular focus on language analytics and build on one or more of the processing frameworks presented before. The more recent LAPPS Grid workbench is based on the generic Galaxy WMS and integrates components across multiple processing frameworks. If two components from the same processing framework are adjacent to each other, they communicate in their native formats, while a small piece of interfacing code called a ‘shim’ is inserted when two adjacent components come from different frameworks. The ‘shim’ then takes care of converting the data before passing it on.

These workbenches are based on different philosophies with respect to use and deployment. Both Argo and WebLicht provide the user with a predefined set of components for text mining. A user cannot currently integrate their own components. These platforms expect that all processing is performed on computing resources which are part of the platform and under the control of the platform providers. Deploying arbitrary custom components on these platforms would present a legal and security risk to the providers and is therefore not appropriate for these platforms. The user, however, only requires a machine capable of browsing the web to use these services, rather than their own high performance computing infrastructure as with other workbenches.

The same is true in principle for the LAPPS Grid with the difference that interested users can actually install their own instance of the LAPPS Galaxy. This instance can then either talk to LAPPS Grid services or to locally deployed components. Also, users can extend such a local installation with new components. Being based on Galaxy, custom components can be installed either manually or through a Galaxy Tool Shed repository.

U-Compare (10) is a standalone application and provides a documented mechanism for the integration of local components. However, there is no explicit support for obtaining additional components from a repository.

GATE Developer and the UIMA Ruta Workbench are locally installed applications. They also support the use of arbitrary custom components compatible with their underlying processing frameworks. GATE components can be installed into the GATE Developer application from external websites hosting GATE component repositories. UIMA Ruta can be used in conjunction with components obtained from Maven repositories.

The way the projects are driven also differs greatly. WebLicht (81) is a part of the CLARIN-D effort, a large-scale infrastructure in Germany and part of the multi-national EU CLARIN effort aiming for a European infrastructure for language resources and technology in the social sciences and humanities. The LAPPS Grid project has a similar goal in the US but is comparably much smaller.

The U-Compare system was superseded by Argo (9) at NaCTeM. The vision of Argo is to create an easy to use but highly functional WMS for the life-sciences community and beyond to engage in a variety of tasks around text mining, including biocuration (82). It provides a powerful mechanism to obtain and process multiple documents in a user-friendly environment. A variety of export options are available to obtain the final results of processing, including type systems tailored to a particular application (83) and web services supporting interoperable formats (84). Argo is accessible and usable via the web, where a large collection of ready-to-use components can be combined by a novice user to build a workflow. NaCTeM have also used Argo as a tool in collaborations, installing separate instances at partner institutions to enable others to benefit from the software. The system has been applied in discovering phenotypes in clinical records (85), implementing state-of-the-art chemical recognition algorithms (86) and semi-automatic curation of disease phenotypes (87).

The GATE framework (42) is mainly developed at the University of Sheffield with partners such as Ontotext. However, it is developed as an open source project hosted on Sourceforge with a public code repository. They also accept code contributions from the community at large. Additionally, there are community-provided repositories of GATE components, such as the Semantic Software Lab at Concordia University in Montréal, Canada.

UIMA Ruta (88) is part of the Apache UIMA project hosted at the Apache Software Foundation. Like all Apache projects, it is an independent volunteer-driven community project providing its software under the liberal conditions of the Apache Software License which suits research and education as well as commercial use. Contributions from the community are welcome.

### General purpose workflow engines

As opposed to a TM specific workflow, many applications exist for the creation of general purpose scientific workflows. These systems provide functionality for reimplementing experiments and for saving, exporting and sharing experimental code which can be easily re-run by other researchers who are interested in the given experimental results. These systems provide a means to build multi-step computational analyses akin to a recipe. They typically provide a workflow editor with a graphical user interface for specifying what data to operate on, what steps to take, and what order to do them in. A general purpose solution can be adapted for use in a TM context by using TM resources if they are available, e.g. see a case study for SADI (89). Although general purpose workflow engines create an internal form of interoperability at the level of the process model, where all components within a workflow will work together, workflow engines from different providers cannot typically be expected to interact. Also, interoperability at the level of the process model does not automatically entail interoperability on the level of the data model, annotation type system, data serialization format, etc. A comparison is given in the Table 13 below.

**Table 13.** A comparison of general purpose workflow engines

Name	Description of modules	License	Example domains	Component creation	Language
ELKI^a^	data mining algorithms; clustering; outlier detection; dataset statistics; benchmarking, etc.	GNU AGPL	Cluster benchmarking	Programming new Java components	Java
Galaxy^b^	genome research; data access; visualization components	AFL 3	Bioinformatics	command line tools	Python
Kepler^c^	Wide variety of components	BSD	Bioinformatics, data monitoring	Java components, R scripts, Perl, Python, compiled C code, WSDL services	Java
KNIME^d^	Univariate and multivariate statistics; data mining; time series; image processing; web analytics; TM; network analysis; social media analysis	GPL3	Business intelligence, financial data analysis	Java, Perl, Python code fragments	Java (Eclipse plugin)
Pegasus^e^	Shell scripts; command line tools	Apache	Astronomy, bioinformatics, earthquake science	Command line	Java, Python, C
Pipeline Pilot^f^	Chemistry; Biology; Materials Modelling; Simulation	Proprietary	Chemicals, Energy, Consumer Packaged Goods, Aerospace	Users cannot create components	C ++
Taverna^g^	Wide variety of components	LGPL	Bioinformatics, astronomy, chemo-informatics, health informatics	WSDL, SOAP and REST services, Beanshell scripts, local Java API, R scripts	Java
Triana^h^	audio, image, signal and text processing; physics studies	Apache	Signal processing	Programming new Java components	Java
SADI^i^	access to the databases and analytical tools for bioinformatics	BSD	Bioinformatics	Web services	OWL, RDF, SPARQL

These can be used for a variety of scientific programming applications, of which one is TM. We have provided some examples of the typical usages of these resources in the table above.

a http://elki.dbs.ifi.lmu.de/

b https://galaxyproject.org/

c https://kepler-project.org/

d https://www.knime.org/knime-analytics-platform

e https://pegasus.isi.edu/

f http://accelrys.com/products/collaborative-science/biovia-pipeline-pilot/

g http://www.taverna.org.uk/

h http://www.trianacode.org/

i http://sadiframework.org/content/

It might be a hard task to select the system that perfectly fits one’s purpose. However, taking into account the unique characteristics of each system will help in the decision process. Initial design purpose of the system is one of the features that have a major effect on the overall usability of such systems. Among the discussed systems, Kepler (90), Pegasus (91) and Taverna (92) are those with the aim of creating a general purpose scientific workflow engine. Thus, it is assumed that they would be the least coupled with any particular domain, and easiest to adapt to new domains. In contrast, ELKI (93), KNIME (94) and Triana (95) were originally created to perform data mining tasks; hence, their power resides in implementing and executing statistical algorithms. Other workflow engines were created for specific domain experiments and later also applied to other domains as well.

The next important classifying feature is the creation of new components for a workflow engine. All of the mentioned tools except Pipeline Pilot (96) and SADI (97) are implemented using Java or Python. This makes them platform independent, and also facilitates the implementation of new components. SADI is not a typical workflow engine, but rather a set of design patterns that help to achieve interoperability and let users combine different tools into a pipeline. It is using the well-known standards of semantic web: each component is a RESTful service communicating using OWL, RDF and SPARQL. Kepler and Taverna also offer direct support for the integration of WSDL services as workflow components. Taverna also supports SOAP and REST services.

In addition to ease of component development, provision of a publicly accessible repository of workflow components is also important. In this aspect, Kepler, Galaxy (98) and Taverna are the only projects that offer a public central repository of components. In contrast, the Kepler system enables the creation of a single executable KAR (Kepler Archive) file of a workflow, which conforms to the JAR (Java Archive) file format. ELKI creates native JAR files.

The deployment model and execution model of a workflow plays a major role in the choice of a workflow engine. In this sense, ELKI, Kepler, Galaxy and Pegasus support executing workflows on a computing grid or cloud. Additionally, fault tolerance of the Pegasus workflow engine is a feature that should not be neglected. This feature would bring major benefits in times where a process intensive workflow fails in the middle of execution.

Another factor to be considered is the community effect: is there a strong developer community who maintains the product and in which communities is the product being used? In this respect, we note that, recently, the LAPPS project and the Alveo project have adopted Galaxy. LAPPS is a project based in the USA that aims at creating a grid-like distributed architecture for NLP. The Alveo project has similar goals and is based in Australia.

As discussed before, choosing the suitable workflow engine is not a trivial task. However, considering general properties of different systems enables a smart decision. It should be noted also that reviewing the already applied domains and example usage scenarios of these workflow engines will be greatly beneficial.

### Discovering tools and services

This section describes the online repositories where tools and services can be discovered. Some of these also contain records for documents and corpora. We have organized the repositories into the following two categories:

1. *Registries*: registries facilitate discovery by maintaining metadata on tools, services and data. However, they do not actually host these, such that downloading or executing them requires the involvement of additional sites. It may not even be possible to access the referenced resources at all, e.g. because they have not been publicly released. Online registries of tools and services that (at least partially) concern text processing are numerous. For each of them, we provide a number of available services, accessibility, supported standards, status and domain. We also include references, when available.
2. *Platforms*: the final set of resources presents platforms that focus on the interaction between language resources and text processing services, i.e. enable running of web services either on data included in the platform or uploaded by the user. They target users with low expertize in technology and try to provide ready-made solutions for processing data rather than discovering resources.

The number of resources that can be discovered or obtained through these sites vary greatly. For example, the CLARIN VLO tends to count each individual document or recording as a separate entry, even if these would otherwise be considered to be part of a collection or corpus. On the other hand, the LINDAT/CLARIN repository has only a single entry for each corpus, irrespective of its size.

The information contained in the repositories can be seen from two perspectives: as a human or as a machine. From the perspective of a user who want to discover tools and services relevant for their task at hand and field of interest the following features may be acceptable: free text metadata, heterogenous forms of formatting and packaging resources (e.g. as separate files, ZIP files, various file formats), the need to authenticate in a web browser, or even the need to send a mail to the resource creator to request access. For automated processing by a machine, the following features are mandatory: controlled vocabularies, the use of standard file and packaging formats, and the ability to obtain a URL to access a resource. Registries presently still target mostly the human user and offer only limited metadata related to programmatic access. As a consequence, it is not straightforward for platforms like LAPPS or ALVEO to make use of these sites as sources for workflow components or for content to be processed.

Most of the sites listed above are based on open source software, often software created by the site maintainers themselves. Thus, it is possible to discover not only if the services are available and being used, but also if they are actively being maintained and or being further developed. We include relevant information about the service status (Running/Closed), about the last update to the service, and a link to the open source code repository in Table 14.

**Table 14.** A comparison of repositories for tools and services that can be redeployed in the text miner's workflow

Title	Available records	Accessibility	Status	Domain	Category
BioCatalogue^a^ (99)	1,184	Open access/use and open registration	Running, last updated in 2015^b^	Life sciences	Registry
Biodiversity Catalogue^c^	71	Open access/use and open registration	Running, last updated in 2015^d^	Biodiversity	Registry
Orbit^e^	89	Open access/registration requires approval	Running, last updated in 2015	Biomedical informatics	Registry
AnnoMarket^f^ (100)	60	Paid for (customers can pay to use any service, third parties can upload their own services and data to sell)	Closed, last updated in 2014^g^	General	Platform
META-SHARE^h^ (20)	more than 2,765	Restricted (anyone can access but addition of new resources requires registering as a META-SHARE member)	Running, last updated in 2016^i^	General	Registry
LRE Map^j^ (21)	3985	Closed (no option to add own resources)	Running, closed source	General	Registry
ALVEO^k^ (101)	34	Open use of services; uploading of services locked	Running, last updated in 2016^l^	General	Platform
Language Grid^m^ (102)	142	Open use of services for non-profit and research; uploading of services for members	Running, last updated in 2015^n^	General	Platform
LAPPS Grid^o^ (43)	45	Open use of services; uploading of services locked	Running, last updated in 2016^p^	General	Platform
QT21^q^	598	Open browsing and use of services, restricted registry	Beta, closed source	General	Platform
LINDAT/CLARIN^r^	1162	Open	Running, last updated 2016^s^	Open	Registry
CLARIN Virtual Language Observatory^t^ (19)	880 915	Open	Running, last updated 2016^u^	Open	Registry

There is a large variation in the size and accessibility of these repositories.

a https://www.biocatalogue.org/

b https://github.com/myGrid/biocatalogue

c https://www.biodiversitycatalogue.org/

d https://github.com/myGrid/biocatalogue

e https://orbit.nlm.nih.gov/

f https://annomarket.com

g https://github.com/annomarket

h http://www.meta-share.eu

i https://github.com/metashare/META-SHARE

j http://www.resourcebook.eu

k http://alveo.edu.au

l https://github.com/Alveo

m http://langrid.org

n http://svn.code.sf.net/p/servicegrid/code

o http://www.lappsgrid.org

p https://github.com/lappst

q http://www.qt21.eu

r https://lindat.mff.cuni.cz/en/

s https://github.com/ufal/lindat-dspace

t https://vlo.clarin.eu/

u https://github.com/clarin-eric/VLO

## Discussion

Despite the obvious advantages of text mining, several obstacles have limited its widespread uptake amongst those in the life sciences who could profit from it most. The first obstacle is a lack of power in the computing resources that underpin text mining software, especially for large-scale processing. The advent of distributed, cloud-based computing has helped to put an end to this issue in recent years. The second obstacle is the prototypical nature of many systems, especially those based on natural language processing techniques, whose designers were faced with adapting general language tools to the particular challenges presented by scholarly communication in the life sciences. While research in the field is ever-on-going, there are now mature, robust tools and systems that achieve results comparable with those of human analysis in many life sciences tasks. The third obstacle, complementing the second, is the lack of suitably annotated data to better understand the types of problem and train supervised machine-learning based systems. Collaboration with domain specialists within the context of community evaluation challenges (103), such as BioCreative (104), BioNLP (105), BioASQ (106) and other annotation efforts (107, 108), has mitigated this lack through the provision of gold standard corpora for certain well-defined competitive text mining tasks designed to advance the state of the art. However, it remains true that a researcher interested in applying text mining to some particular research question concerning a particular sub-domain may be faced with a lack of some trained tool or annotated corpus that would hamper their efforts. We have seen throughout this paper, however, how other aspects of text mining can help reduce the time and effort it would otherwise cost to fill such a gap. The fourth obstacle, again somewhat related to the second, is lack of interoperability. This presents itself in various guises. For example, a tool might split a text into tokens and then tag it for part of speech, but such a black box combination of processes means that one could not, for example, use a different tokenizer better suited to the task in hand, say, for tokenization of chemical compounds. Such issues have gradually become less important, due to a general move in software engineering towards component-based systems. However, natural language processing and text mining are further affected by interoperability issues at the linguistic and indeed conceptual level. A simple example will suffice to illustrate: a researcher finds two tools, one that recognizes the names of bio-entities in text and another that extracts relations among bio-entities. However, the entity types (labels) that the first produces are not the same as those that the relation finder expects to find in its input: there may be no intersection or a partial one; even if there is an intersection in terms of names, there may be none in terms of what entities they refer to. This lack of interoperability has been a major blocking factor for developers and users. Fortunately, in recent years, there has been much progress made on standardization and normalization in the field, such that interoperability is much enhanced. Although interoperability is not a totally solved problem, recent initiatives (shown below) have yielded benefits for both developers and users alike. The fifth obstacle is that access to content for text mining is frequently limited because of legal reasons (109). Publishers of non-open access journals usually require TM researchers to negotiate a licence agreement for every research project and impose several restrictions, e.g. non-commercial use (110). This has been alleviated by exceptions for TM introduced in several countries, which allow researchers to automatically mine the content they have lawful access to. Lack of such regulations in some areas, e.g. the European Union, significantly hampers data mining research (111).

This study is a part of the efforts of the on-going OpenMinTeD^17^ project to build an interoperable text and data mining (TDM) infrastructure, which could help to relieve some of these obstacles. As such, we have tried to promote interoperability standards throughout this report where possible. Interoperability is a topic which has already been discussed and studied at length over multiple large research projects. The CLARIN Project (19) produced a review of accepted standards which were designed to promote interoperability in multiple fields. The META-NET project (112) produced a language resources sharing and discovery infrastructure, META-SHARE (20), along with associated metadata standards (11). The FLaReNeT project, aiming to create a European strategy for the area of language processing and resources, prepared an assessment of the current standards’ landscape (113). Other on-going efforts include the Research Data Alliance^18^ initiative for promoting openness in research data sharing, which has several working groups which are interested in interoperability and FutureTDM^19^, which tries to assess and reduce legal and policy barriers for the growth of the text mining field. Another source of increasing interoperability in TDM are large ecosystems, such as UIMA and GATE, supporting open standards and attracting a lot of users, who are also able to contribute their own components.

In this study we aimed to give an accurate account of the landscape of available text mining resources for biocuration, but clearly our approach has its limitations. The field has been divided into areas and subareas corresponding to tasks in a TM application and we selected the most important and representative resources in each. We could not include every possible item as there is a long tail of resources created for a particular problem, often within a single project, and then abandoned with little or no support or documentation. We have shown that there are at least several options to choose from at every step of the text mining process, which makes it possible to construct a working end-to-end application. The choices a user makes could be motivated by factors other than core functionality, e.g. resource interoperability, usage of open standards, prior conventions or what has already been successfully applied in the target domain.

We have focused on text mining for the life sciences in this study. However, text mining is also growing in many other areas. We have chosen not to speak about these in this survey, but instead leave a more general overview of the text-mining field to further work. Our decision to take this focus is wholly appropriate, as the life sciences is the most common domain in text-mining research (114). The life sciences has very well-developed terminological resources which make text mining easier and many publications are published in open-access journals—making them accessible for text mining. There is also a great need for text mining in the life sciences, as evidenced by the now infamous ‘data deluge’. Even a researcher in a minor sub-field is expected to keep up with increasingly large volumes of new publications. Fortunately, the techniques that we have discussed in this report are transferable to other domains. Many of the repositories that we have discussed are not solely focused on the life sciences but also contain TM resources for other appropriate domains.

Throughout this study, we have tried to give prominence to those resources which are the result of efforts towards interoperability. We have seen a wide range of interoperability throughout the report. Some sections (e.g. Mechanisms used for the identification of resources, Annotation Models, Formats for Knowledge Resources, General Purpose Workflow Engines) relate to areas with comparatively low levels of interoperability. In these areas, there is little uptake of existing standards, or maybe no standards altogether. Other areas exhibit a high degree of potential for interoperable systems (e.g. Metadata schemata and profiles, Vocabularies and ontologies for describing specific information types and Text mining workflow management systems). These areas may have multiple competing standards which each allow a user to build and access resources which are easy to connect to pre-existing code due to their implementation of existing interoperability standards. It can sometimes be the case that standards exist, but they are not used because the community is not aware of them. We hope that this study goes some way towards addressing that gap. To this end, we have promoted interoperability standards wherever possible alongside a discussion of the virtues of integrating resources with these standards. There are some cases where it may not be appropriate for a user to implement interoperability standards—e.g. a closed ecosystem, rapid prototyping or while integrating with third-party tools. However, a user should be able to consciously choose not to implement an interoperability standard, rather than not knowing about its existence in the first place.

We have presented a set of relevant repositories at the end of each section. These are intended to help guide the reader to find a wider set of resources than those we have mentioned in this paper. The repositories are kept current and so by looking at these repositories the user can find relevant and up-to-date resources. The lists of repositories are not meant to be binding or comprehensive, but instead are intended as a useful list of places for the reader to get an idea of what is on offer. It will be beneficial in most cases for the user to search for repositories which are related to the type of work that they are doing. If none can be found, then the reader may wish to consider starting their own repository, implementing some of the metadata standards which we have previously discussed. When browsing a repository, the user should consider questions such as: ‘What other kinds of resources are typically stored in this repository?’, ‘What types of metadata are used to store resources?’ and ‘How easy is it to upload new resources?’. We have tried to equip the reader throughout this report to be able to answer these questions for themselves.

A recent study by Thompson *et al.* (115) may serve as an example of combining all of the elements of text mining within a single project. The goal was to analyse medical vocabulary from a historical perspective, observing how certain terms and concepts appear, transform and wither across the years. The authors started by acquiring content from the British Medical Journal archive, which is accessible via CrossRef (see ‘Metadata schemata and profiles’ section), and London Medical Officer of Health reports, which are downloadable in multiple formats. Next, the texts were manually annotated with medical entities and saved in the BRAT format (see ‘Annotation models’ section). To create a time-sensitive inventory of medical terms, the authors both implemented an automatic method based on distributional semantics and employed a thesaurus aggregating over 150 terminological resources (see ‘Useful knowledge resources’ section). The obtained corpus has been used to study the performance of named entity and event recognition techniques, implemented in the Argo workflow manager (see ‘Text mining workflow management systems’ section) using readily-available components. Finally, both the annotated corpus and the term inventory encoded in OBO format (see ‘Formats for knowledge resources’ section) were published in the META-SHARE repository (see ‘Language resources repositories’ section). Employing open standards, formats and services for publishing annotated content, created vocabularies and workflows, makes it more likely that such a study will be useful for related research projects.

The on-going OpenMinTeD project is at the forefront of promoting text and data mining amongst the communities that need it most. The project is currently working on several fronts to further the cause of text and data mining. First a platform will be produced, which will allow a novice TM user to come and experiment with some standard tools and their own data. Second, a set of flagship applications will be developed using the platform to demonstrate the power of TM and promote TM within the communities that the applications are developed for. Third, the project will provide a set of interoperability guidelines which will allow third party applications to integrate with the platform. This will make the platform a focal point for new technology. Application developers will benefit from implementing the interoperability specifications as their tool will gain access to a wide market of users. Finally, the project will provide training and community engagement events to educate and equip users who may not have the technical expertise to use TM within their own research. The audience of this paper should also make themselves aware of such efforts, as they are designed to reduce the difficulty encountered by the novice text miner.

In this report, we have covered a wide variety of topics, from where to find publications for text mining in ‘Content discovery’ section, through how resources are encoded in ‘Knowledge Encoding’ section and finally how to bring resources and components together in a text mining workflow in ‘Tools and services’ section. We have equipped the reader with all the knowledge they need to make informed choices about the resources that currently exist in the field. The final decision of how to use these resources to extract useful information from their data rests with the reader.

## Footnotes

1 http://www.ncbi.nlm.nih.gov/pubmed

2 https://www.nlm.nih.gov/bsd/stats/cit_added.html

3 http://www.niso.org/standards/z39-96-2015/ (November 2015) NISO JATS Version 1.1 (ANSI/NISO Z39.96-20151)

4 http://www.ncbi.nlm.nih.gov/pmc/tools/oai/

5 https://www.openaire.eu/

6 http://www.rioxx.net/profiles/v2-0-final/

7 http://www.niso.org/apps/group_public/project/details.php?project_id=117

8 http://www.tei-c.org/Guidelines/P5/

9 http://www.lrec-conf.org/

10 https://www.w3.org/TR/vocab-dcat/

11 https://archive.org/services/purl/

12 http://www.ietf.org/rfc/rfc3650.txt

13 http://www.doi.org/

14 http://orcid.org/

15 http://www.omg.org/spec/XMI/

16 http://www.ldoceonline.com/dictionary/catalogue_1

17 http://openminted.eu/

18 https://rd-alliance.org/

19 http://project.futuretdm.eu/
